# Spatial UBE2N protein expression indicates genomic instability in colorectal cancers

**DOI:** 10.1186/s12885-019-5856-1

**Published:** 2019-07-18

**Authors:** Timo Gemoll, Elena Miroll, Oliver Klein, Annette Lischka, Murat Eravci, Christoph Thorns, Jens K. Habermann

**Affiliations:** 1 Section for Translational Surgical Oncology and Biobanking, Department of Surgery, University of Lübeck and University Medical Center Schleswig-Holstein, Campus Lübeck, Lübeck, Germany; 2 Berlin-Brandenburg Center for Regenerative Therapies, Charité – Universitätsmedizin Berlin, Berlin, Germany; 3 Institute of Chemistry and Biochemistry, Biochemistry, Freie Universität Berlin, Berlin, Germany; 4 Institute of Pathology, University Medical Center Schleswig-Holstein, Campus Lübeck, Lübeck, Germany; 5 Interdisciplinary Center for Biobanking-Lübeck (ICB-L), University of Lübeck, Lübeck, Germany

**Keywords:** Imaging mass spectrometry, Genomic instability, Aneuploidy, UBE2N

## Abstract

**Background:**

One major hallmark of colorectal cancers (CRC) is genomic instability with its contribution to tumor heterogeneity and therapy resistance. To facilitate the investigation of intra-sample phenotypes and the de novo identification of tumor sub-populations, imaging mass spectrometry (IMS) provides a powerful technique to elucidate the spatial distribution patterns of peptides and proteins in tissue sections.

**Methods:**

In the present study, we analyzed an in-house compiled tissue microarray (*n* = 60) comprising CRCs and control tissues by IMS. After obtaining protein profiles through direct analysis of tissue sections, two validation sets were used for immunohistochemical evaluation.

**Results:**

A total of 28 m/z values in the mass range 800–3500 Da distinguished euploid from aneuploid CRCs (*p* < 0.001, ROC AUC values < 0.385 or > 0.635). After liquid chromatograph-mass spectrometry identification, UBE2N could be successfully validated by immunohistochemistry in the initial sample cohort (*p* = 0.0274, ROC AUC = 0.7937) and in an independent sample set of 90 clinical specimens (*p* = 0.0070, ROC AUC = 0.6957).

**Conclusions:**

The results showed that FFPE protein expression profiling of surgically resected CRC tissue extracts by MALDI-TOF MS has potential value for improved molecular classification. Particularly, the protein expression of UBE2N was validated in an independent clinical cohort to distinguish euploid from aneuploid CRCs.

**Electronic supplementary material:**

The online version of this article (10.1186/s12885-019-5856-1) contains supplementary material, which is available to authorized users.

## Background

Colorectal cancer (CRC) is the third most frequent type of cancer with a worldwide incidence of 1.4 million people [1]. Nearly every CRC arises from three pathways, namely, chromosomal instability (CIN), microsatellite instability (MSI) and CpG island methylation (CIMP) [2–4]. While the MSI pathway is defined by failures in the DNA mismatch repair system (MMR) and an increased rate of mutations [5], the CIMP pathway is characterized by the altered methylation of genes, which results in modified transcription, e.g., the silencing of tumor suppressor genes [2]. In contrast, the CIN pathway displays a high rate of chromosome segregation errors that lead to aneuploidy and affects more than 80% of all CRCs [6]. Chromosomal rearrangements and deviations from the euploid chromosome number seem to contribute to intratumor heterogeneity being increasingly recognized as a common phenomenon across human carcinomas [7]. Intratumor heterogeneity is histologically indistinguishable at the microscopic level [8] but is thought to result in unique molecular subtypes that drive tumor progression and determine the disease outcome of the patient [9, 10]. In this context, aneuploidy remains underestimated when analyzing mechanisms of tumor progression and relapse and when used as a parameter for individualized medicine. Several studies have revealed that CIN onset precedes subclonal expansion and metastatic dispersion in several cancers and shares the same chromosomal aberrations between intratumoral regions [11–13]. Hence, it is of absolute importance to elucidate aneuploidy-associated colorectal carcinogenesis respecting its role of intratumor heterogeneity for individualized medicine.

Thus far, the emphasis of research into molecular tumor heterogeneity has been on the genomic and transcriptomic levels, including evolutionary divergence between primary tumors and metastatic outgrowths [14]. Traditionally, spatial heterogeneity in the tissue samples has been limited to microscopy-based assessment, e.g., immunofluorescence and fluorescence in situ hybridization. These targeted techniques are limited (i) to very low throughput, (ii) to few molecules, (iii) in quantifying expression levels and (iv) in comparing heterogeneity between samples. On the proteome, lipidome and metabolome level, matrix-assisted laser desorption/ionization (MALDI) imaging mass spectrometry (IMS) has emerged and combines de novo identifications with unsupervised and unlabeled spatial resolution. By correlating molecular information with histology at spatial resolution, MALDI-IMS enables the analysis of both the relative abundance and distribution of proteins. In this context, we could already show that ploidy measurements and MALDI-IMS using fresh frozen material can identify prognostic markers in CRC, e.g. thymosin beta-4 [15]. However, routine diagnostics are based on FFPE material [16]. Thus, we now aimed to identify prognosis-associated proteins in FFPE material by MALDI-IMS. Depicted targets could provide new insights into genomic instability, tumor heterogeneity and treatment options for individualized medicine.

## Methods

### Patient cohorts

For the protein profiling by IMS and the first validation by IHC, one tissue microarray (TMA) was compiled containing 40 human colorectal carcinomas (CRC) and 20 normal human colorectal mucosa samples (training TMA, Table 1) representing 20 patients in total.

**Table 1:** The clinical characteristics of colon cancer samples used for tissue microarray (TMA) analysis (training set) by means of MALDI-imaging and immunohistochemistry

Clinical parameter	Samples of adjacent non-neoplastic mucosa	Samples of CRCs^a, b^
Sex [male/female]	14/6	20/20
Age [years]	64.5	66.2
Ploidy [euploid/aneuploid]		20/20
T-status [1/2/3/4]		0/4/28/8
N-status [0/1/2]		20/10/10
M-status [0/1]		36/4
Grading [1/2/3]		0/28/12
Survival status [alive/dead]		18/22
Survival [months]		77.4

^a^ Two samples could not be included in the SPTBN1 staining

^b^ Three samples could not be included in the UBE2N staining

A second TMA of 60 human CRCs and 30 normal human colorectal mucosa samples was used as a subsequent independent validation set for immunohistochemistry only (validation TMA, Table 2). For both TMAs, all of the colorectal cancer samples were equally subdivided into carcinomas with lymph node positive and negative metastasis, euploid and aneuploid cancers, UICC I/II and UICC III/IV cancers and patients with a survival of less and more than 60 months. Patients with palliative treatment (R1 resection), survival < 30 days after surgery, and neoadjuvant radiotherapy for rectal tumors were excluded. Patients matching the Amsterdam II-criteria for the hereditary-non-polyposis-colorectal-cancer (HNPCC) syndrome were also excluded. The study was conducted in adherence to the approval by the local Ethical Committee of the University of Lübeck (#07–124).

**Table 2:** Clinical characteristics of colon cancer samples used for tissue microarray (TMA) analysis (validation set) by means of immunohistochemistry

Clinical parameter	Samples of adjacent non-neoplastic mucosa	Samples of CRCs^a^
Sex [male/female]	16/14	30/30
Age [years]	56.7	68.7
Ploidy [euploid/aneuploid]		30/30
T-status [1/2/3/4]		2/11/39/8
N-status [0/1/2]		30/23/7
M-status [0/1]		60/0
Grading [1/2/3]		3/41/16
Survival status [alive/dead]		33/27
Survival [months]		68.1

^a^ Seven samples could not be included in the UBE2N staining

### Genomic instability assessment

Genomic instability was assessed by nuclear DNA ploidy measurements by means of image cytometry using Feulgen stained imprints as described previously [15, 17]. Briefly, images of at least 500 nuclei were selected manually to create DNA ploidy histograms using the ACAS imaging system (Ahrens ACAS, Hamburg, Germany). Histograms were classified in euploid and aneuploid cell populations according to Auer [17]. Hereby, DNA histograms of types I (diploid), II (tetraploid), and III (diploid-proliferating) were characterized as euploid cell populations. All DNA histograms were evaluated by three independent investigators who were unaware of the clinical and histopathological data of the patients.

### MALDI-imaging mass spectrometry experiments

Prior to trypsin and matrix application, optical images of the training TMA were obtained using a standard flatbed scanner (ScanMaker 9800X2, Microtek, Germany). A total of 200 μl of the trypsin solution (20 μg, 20 mM ammonium bicarbonate/acetonitrile 9:1) was applied directly onto the section using an automated spraying device (ImagePrep, Bruker Daltonik, Germany) according to the manufacturer’s protocol, with 39% power ± 0% modulation. Tissue incubation with the trypsin solution was performed for 3 h at 37 °C in a moist chamber. Following trypsinization, the matrix solution (7 g/l α-cyano-4-hydroxycinnamic acid in 50% acetonitrile and 1% trifluoroacetic acid) was applied in ImagePrep using the manufacturer’s protocol with 15% power ± 40% modulation [18].

MALDI-IMS data acquisition was performed as previously described [19]. Briefly, peptides were detected using an Autoflex III MALDI-TOF/TOF with flexControl 3.0 and flexImaging 3.0 software (Bruker Daltonik; Germany) in a positive ion reflector mode. After application of the measuring settings in flexControl (detection range of m/z 800–3500, 200 laser shots per spot, a sampling rate of 0.5 GS/s, raster width of 80 μm), an external calibration was conducted using a peptide calibration standard (Bruker Daltonik, Germany) which was spotted next to the tissue section area. Spectra processing was performed in flexAnalysis 3.0 (Bruker Daltonik) with spectra smoothing and baseline subtraction. Evaluated tissue sections were subsequently treated with 70% ethanol to remove the matrix and stained with hematoxylin and eosin (H&E) [20].

Investigation of MALDI-IMS raw data was performed after conversion to the SCiLS SL file format, baseline removal and total ion count (TIC) preserving using SCILS Lab (SCiLS Lab-2015b, Bremen, Germany). Peak findings and alignment were created by a standard segmentation pipeline. Regions containing only mucosa or carcinoma tissue were marked based on morphology to limit the analysis to relevant parts of the tissue samples. A receiver operating characteristic (ROC) analysis was performed to identify discriminating properties (peptides) between the different tumor types or normal mucosa tissue. Univariate hypotheses tests (Wilcoxon rank sum test) were used to ensure statistical significance (*p* < 0.001) of the m/z values with ROC AUC values < 0.385 or > 0.635. This approach was performed for the groups containing only mucosa vs. carcinoma tissue and on 5-year-survival positive vs. negative, nodal status N0 vs. N1 + 2, UICC status I + II vs. III + IV and euploid vs. aneuploid data.

### Protein identification

To identify the m/z values, complementary protein identification was performed on adjacent training TMA sections through a “bottom-up”-nano liquid chromatography (nLC) - MS/MS approach. Briefly, after digestion, the peptide samples were desalted by solid phase extraction (SPE) using C18 stage tips [21]. Desalted peptide mixtures were separated by reverse-phase chromatography using a Dionex Ultimate 3000 nanoLC on in-house manufactured 25 cm fritless silica microcolumns with an inner diameter of 100 μm. The columns were packed with ReproSil-Pur C18-AQ 3 μm resin (Maisch GmbH, Ammerbuch-Entringen, Germany). The peptides were separated on a 5–60% acetonitrile gradient with 0.1% formic acid at a flow rate of 350 nL/min for 90 min. Eluting peptides were ionized on-line by electrospray ionization and transferred into an LTQ Orbitrap Velos mass spectrometer (Thermo Fisher Scientific, Bremen, Germany). The LTQ-Orbitrap was operated in the positive mode to simultaneously measure full-scan MS spectra (from m/z 300–1700) in the Orbitrap analyzer at resolution *R* = 60,000 following isolation and fragmentation of the twenty most intense ions in the LTQ part by collision-induced dissociation.

Protein identification was performed with the MaxQuant software package (version. 1.3.0.5 / Max-Planck-Institute of Biochemistry, Martinsried, Germany). Initial maximum precursor and fragment mass deviations were set to 7 ppm and 0.5 Da, respectively. Methionine oxidation/acetylation of the peptide N-termini and cysteine carbamidomethylation were set as variable and fixed modification, respectively, for the search. Furthermore, the enzyme specificity was set to trypsin, and a maximum of two missed cleavages was allowed for the search. The target-decoy-based false discovery rate (FDR) for peptide and protein identification was set to 1% for peptides and proteins, and the minimum peptide length was set to seven amino acids. The precursor mass tolerance was set to 20 ppm. The mass tolerance for fragment ions was set to 0.5 Da. MALDI-IMS m/z values required the accordance of more than one peptide (mass differences < 0.9 Da) of the LC-MS/MS reference list to subsequently correctly assign the corresponding protein [22].

### Immunohistochemistry

For validation studies of the IMS results by immunohistochemistry, all TMA-sections were deparaffinized in xylene and hydrated in a graded alcohol series following by antigen unmasking in a citric acid buffer. Incubation with methanol was performed to quench endogenous peroxidase activity. After blocking with diluted horse serum, primary antibodies against SPTBN1 (1:500, monoclonal, #EPR5869, Origene) and UBE2N (1:1500, monoclonal, #D2A1, Cell Signaling) were applied overnight at 4 °C. Staining was performed using the Vectastain universal elite ABC kit (Vector, USA) and the chromogens DAB or AEC as the peroxidase substrate (DAKO, USA) according to the manufacturer’s instructions. Representative histological regions on each slide were scanned by digital microscopy (Pannoramic DESK, 3D Histech, Budapest, Hungary) and assessed quantitatively by Image Scope (v12.1, Aperio, Vista, USA) using the supplied positive pixel count algorithm v9.1. Five different areas per TMA-core were averaged. Immunopositivity of the molecular markers was collected as continuous variables ranging from 0 to 1 as described earlier [15]. One senior pathologist (C.T.) reviewed all of the slides after H&E staining.

### Statistical analyses

The Mann-Whitney U test was performed to test the staining differences with clinical parameters (significance level of 5%). For immunohistochemistry, duplicated TMA-cores per case were averaged. The associations between the clinical characteristics of the colorectal cancer patients and UBE2N protein expression were tested using a Pearson correlation test and ROC.

## Results

### MALDI-IMS and statistical evaluation

A total of 27 colorectal carcinoma (CRC) and 17 normal mucosa tissues could be evaluated by TMA based MALDI imaging. Within the mass range from 800 to 3500 Da, no m/z values indicating differential expression were obtained in the UICC I + II vs. UICC III + IV, positive 5-year-survival vs. negative 5-year-survival or N0 vs. N1 + 2 comparison. However, 29 m/z values discriminated normal mucosa from the CRC areas, and 28 m/z values distinguished euploid from aneuploid CRC region of interests (ROIs) (Table 3).

**Table 3:** Differential expression m/z values (*p* < 0.05) between euploid and aneuploid CRCs

More highly expressed in euploid CRCs	More highly expressed in aneuploid CRCs		
m/z value	ROC AUC	m/z value	ROC AUC
806.466	0.650	3203.604	0.354
959.544	0.660	3244.229	0.354
960.516	0.665	3252.385	0.354
1043.533	0.667	3286.312	0.354
1118.661	0.665	3366.631	0.353
1120.820	0.661	3380.416	0.355
1203.898	0.684	3383.055	0.351
1204.806	0.683	3394.636	0.354
1301.943	0.678	3403.794	0.354
1358.990	0.647	3415.411	0.353
1377.954	0.653	3416.329	0.345
1384.045	0.648	3417.247	0.343
1387.031	0.651	3418.063	0.354
		3444.847	0.354

*ROC AUC* Receiver operating characteristic area under curve

### Identification of proteins

To identify m/z values of interest, we performed a corresponding bottom-up LC-MS/MS approach with adjacent tissue sections from the training TMA-set. All identified peptides were compared with the observed m/z values from the MALDI-IMS experiment and revealed the proteins Alpha-Enolase 1 (ENO1), Histone H2A type 1-B/E (HIST1H2AB), Spectrin beta chain, non-erythrocytic 1 (SPTBN1), Transgelin-2 (TAGLN2), and Ubiquitin-conjugating enzyme E2 N (UBE2N) with more than one assigned peptide as being identified (Additional file 1: Figure S1 & Table 4).

**Table 4:** Identified protein candidates in the euploid vs. aneuploid comparison. IMS, imaging mass spectrometry; LC-MS/MS, liquid chromatograph-mass spectrometry

IMS [m/z, H^+^]	LC-MS/MS [m/z, H^+^]	Delta [Da]	Protein name	Accession UniProt	Regulation [euploid to aneuploid]
959.544	959.545	−0.001	Alpha-Enolase	ENOA_HUMAN	down
1118.661	1118.665	−0.004			down
**959.544**	**959.513**	**0.031**	**Spectrin beta chain, non-erythrocytic 1**	**SPTB2_HUMAN**	**down**
**1203.898**	**1203.593**	**0.305**			**down**
1203.898	1203.633	0.265	Transgelin-2	TAGL2_HUMAN	down
1384.045	1383.613	0.432			down
**1043.533**	**1043.635**	**−0.102**	**Ubiquitin-conjugating enzyme E2 N**	**UBE2N_HUMAN**	**down**
**1203.898**	**1203.593**	**0.305**			**down**

The targets for subsequent immunohistochemical validation are presented in bold

### Histological and Immunohistochemical validation

**Fig. 1:**
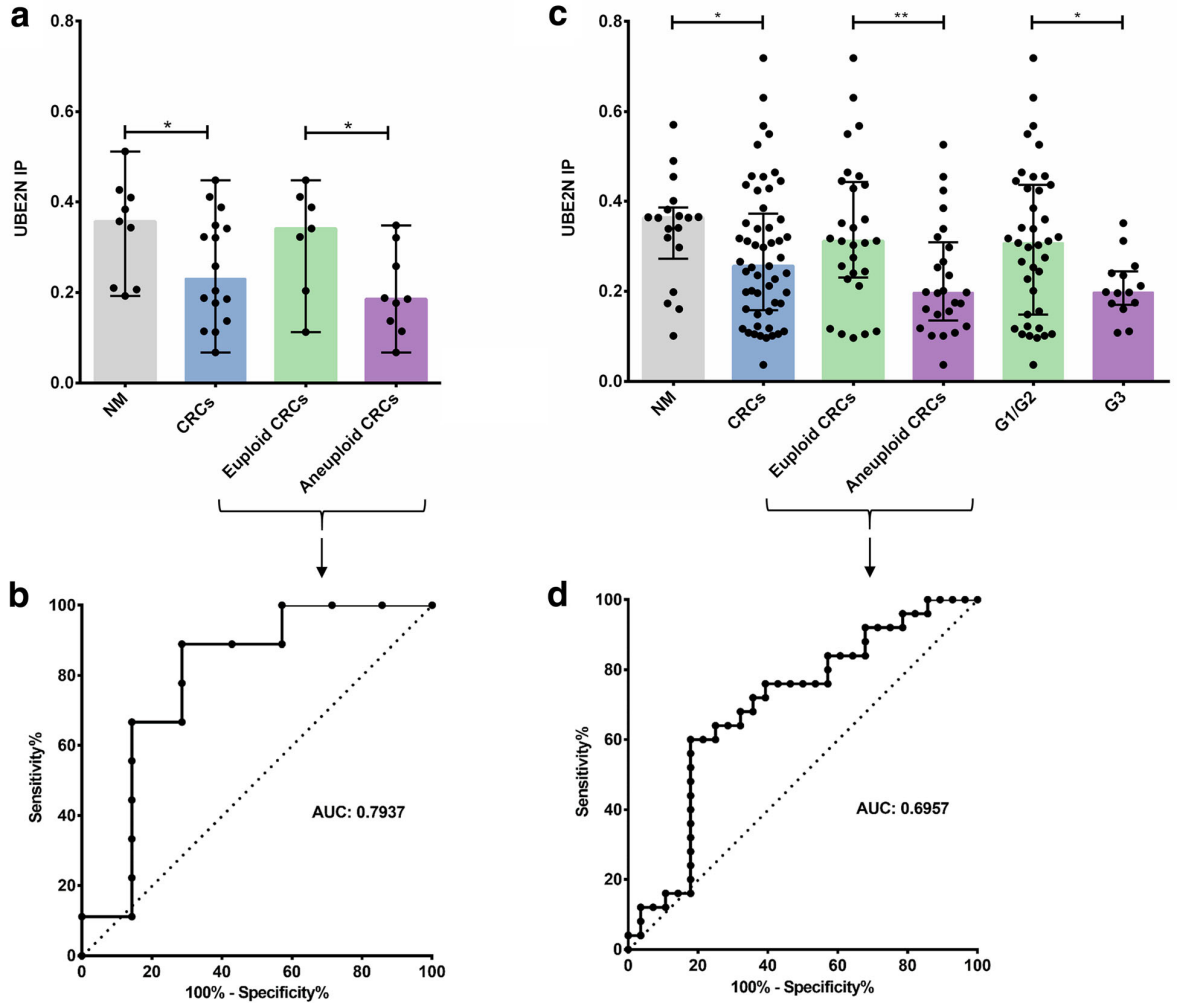
Tissue-microarray-based immunohistochemical evaluation of UBE2N by means of image scope comparing normal mucosa vs. CRC and euploid vs. aneuploid CRCs in the training (**a**, **b**) and validation TMA set (**c**, **d**). ROC curves (**b**, **d**) represent good discriminative power to distinguish between euploid and aneuploid CRC (**0.001 < *P* < 0.01, *0.01 < *P* < 0.05). NM, normal mucosa; CRC, colorectal cancer; IP, immunopositivity; ROC AUC, Receiver operating characteristic area under curve

Based on the results of the discovery protein profiling by IMS and the biological function, we analyzed the identified proteins SPTBN1 and UBE2N using one dependent (training TMA) and one independent (validation TMA) patient cohort. For cytoplasmic UBE2N, the immunohistochemical validation confirmed the MALDI-IMS findings in the training TMA: Immunopositivity (IP) was significantly stronger in the euploid (median 0.3417, 95%CI 0.2072–0.4293) than in the aneuploid CRCs (median 0.1854, 95%CI 0.1275–0.2716, *p* = 0.0274) and showed a higher intensity in the normal mucosa (median 0.3575, 95%CI 0.2517–0.4242) than in CRCs (median 0.2309, 95%CI 0.1881–0.3149, *p* = 0.0284, Fig. 1a). Additionally, ROC analysis appeared to have good discriminating power to differentiate euploid from aneuploid CRCs (ROC AUC 0.7937, Fig. 1b).

Subsequent validation in an independent patient cohort (validation TMA; 18 samples of normal mucosa, 28 euploid CRCs, and 25 aneuploid CRCs) confirmed the increased UBE2N expression in euploid compared to aneuploid CRCs (median 0.3122, 95%CI 0.2687–0.3950 vs. median 0.1973, 95%CI 0.1769–0.2785, *p* = 0.0070) and in normal mucosa compared to tumor tissue (median 0.3637, 95%CI 0.2776–0.3949 vs. median 0.2562, 95%CI 0.2405–0.3250, *p* = 0.0374, Fig. 1c). The ROC curve demonstrated good discriminative power also in the validation set to predict euploid and aneuploid CRCs (ROC AUC 0.6957, Fig. 1d). Evaluating the remaining clinical parameters, only the differentiation status of tumors showed significant differences (G1/2 median 0.3077, 95%CI 0.2551–0.3633 vs. G3 median 0.1978, 95%CI 0.1697–0.2483, *p* = 0.0267, Fig. 1c). No significant differences existed between the UBE2N-negative and UBE2N-positive groups with respect to age (64 vs. 69 years, *p* = 0.497), tumor stage (*p* = 0.516), dichotomized tumor stage (*p* = 0.744), lymph node stage (*p* = 0.540), survival status (*p* = 0.337) or follow-up (*p* = 0.446). Overall survival with respect to UBE2N expression was not significant but showed a trend of being better for UBE2N-positive patients. The discriminative power proved useful after combining immunohistochemical data of the training and validation TMA (ROC AUC 0.71314, Additional file 1: Figure S2). Exemplary stainings are presented in Additional file 1: Figure S3.

The evaluation of the training TMA and SPTBN1 cytoplasmic IP revealed no statistical significance in the euploid (median 0.8401) vs. aneuploid (median 0.7187) comparison (*p* = 0.0905). Conversely to the IMS results, a trend for higher expression in the tumor tissue (median 0.7568) compared to the normal mucosa (median 0.7047) was observed (*p* = 0.0821; Additional file 1: Figure S4).

## Discussion

Therapy response is strongly affected by intra-tumor heterogeneity with distinct molecular subclones. While genome and transcriptome analysis have been proved to be of high value for systems medicine-based therapy prediction, proteome evaluations will complement the disease phenotype fingerprint, particularly in the context of spatial resolution. The identification of proteomic markers is thus urgently needed to define CRC molecularly subclones towards precision medicine. Since aneuploidy does cause tumor heterogeneity which in turn drives tumor differentiation, tumor progression and disease outcome [10, 23–27], it is of great importance to discover markers of genomic instability as potential therapeutic targets. In particular, the characterization of genomic instability for immunotherapy options in colorectal cancer, which based on patients with DNA mismatch repair-deficiency or microsatellite instable tumor cell populations, could play an essential role [28, 29].

This study proved that tissue-based proteomic profiling by MALDI imaging is a valuable tool to identify protein patterns of FFPE material that objectively predict genomic instability in CRC. We identified SPTBN1 (spectrin beta chain, non-erythrocytic 1) and UBE2N (ubiquitin conjugating-enzyme E2 N) using MALDI-IMS and LC-MS/MS to be differentially expressed between euploid and aneuploid samples. Furthermore, the expression difference of UBE2N was validated by immunohistochemistry on two independent TMAs comprising 150 samples in total. This study is the first to show that FFPE-based protein profiling by MALDI imaging is able to identify protein patterns that predict the ploidy status of a given sample. With the impact of aneuploidy on tumor heterogeneity, MALDI imaging clearly shows its ability to be of high clinical value in the future.

UBE2N is a ubiquitin-conjugating enzyme performing K63-linked protein polyubiquitination that creates most ubiquitin chains [30]. UBE2N functions as a heterodimer, binding the two non-catalytic E2-like partner proteins Mms2 and Uev1A in the cytoplasm [31]. While the UBE2N-Uev1 interaction is required for the cellular response to both bacterial and viral infections, UBE2N and Mms2 redistribute to the nucleus upon DNA damage and lead to assemblies of complexes within the RAD6 pathway of DNA repair, thus preventing genomic instability and cancer [31–33]. Alternatively, recent studies have demonstrated that a loss of cytoplasmic UBE2N attenuates NF-κB activation and results in the accumulation of Wingless (Wnt) targets, e.g., β-catenin [34–36]. In turn, elevated levels of β-catenin may lead to a deregulation of adenomatous polyposis coli (APC) that could contribute indirectly to defects in cell migration, chromosome segregation and genomic instability [37, 38]. Accumulation of β-catenin in enterocyte precursors could further lead to the retention of a stem cell phenotype, which eventually leads to the formation of a polyp [39]. Recently, UBE2N appears to act with several E3 ligases which contribute to uncontrolled proliferation and genomic instability when deregulated [40].

Therefore and with the now identified and validated decreased levels of cytoplasmic UBE2N in aneuploid compared to euploid CRCs and normal tissue, low expression levels of UBE2N are in line with its molecular function. Irrespective of its binding partner, a low cytoplasmic level of UBE2N could be explained by the consequence of genomic instability (as a repair mechanism) and/or by the cause of the tumor to activate the NF-κB-associated progression of cancer. In this respect, patients with aneuploid tumors could eventually benefit from the UBE2N inhibitor NSC697923.

Interestingly, low UBE2N expression was additionally correlated with highly dedifferentiated tumors. Assuming that aneuploidy and tissue differentiation lead to increased tumor heterogeneity, UBE2N might be a mere reflection of a possibly decreased therapy response.

## Conclusion

Our data show that using routinely available FFPE tumor tissues, protein profiles can be used to accurately classify genomically stable (euploid) and unstable (aneuploid) tumor types reflecting different tumor biology, prognosis, and therapy resistance. On that basis, UBE2N was identified, validated and discussed as a factor that maintains genomic stability and plays an important role in colorectal cancer development in close relation to β-catenin, APC and Wnt signaling.

## Additional file

Additional file 1: **Figure S1.** MALDI imaging intensity box plots of identified peptides for UBE2N (m/z 1043.635 & m/z 1203.593; top) and SPTBN1 (m/z 959.513 & m/z 1203.593 bottom). Intensity plots of the two identified peptides UBE2N and SPTBN1 detected differentially regulated by MALDI imaging. **Figure S2.** Tissue-microarray-based immunohistochemical evaluation of UBE2N by means of image scope comparing normal mucosa vs. CRC and euploid vs. aneuploid CRCs after combining data of the training and validation TMA set. Bar plots and ROC curve of immunhistochemical data of UBE2N after combining data of the training and validation TMA set. **Figure S3.** Exemplary immunohistochemical UBE2N stainings of the TMA validation set**.** Images are presented as an overview (A) as well as an image section (B). Overview of exemplary immuohistochemical images of UBE2N. The images present results from the TMA validation set. **Figure S4.** Tissue-microarray-based immunohistochemical evaluation of SPTBN1 by means of image scope comparing normal mucosa vs. CRC and euploid vs. aneuploid CRCs in the training and validation set. Bar plots of immunhistochemical data of SPTBN1. The images present results from the TMA validation set. (DOCX 21654 kb)

## Data Availability

The datasets used and/or analysed during the current study are available from the corresponding author on reasonable request.

## Abbreviations

CIMP: CpG island methylation
CIN: Chromosomal instability
CRC: Colorectal cancer
FDR: False discovery rate
H&E: Hematoxylin and eosin
HNPCC: Hereditary-non-polyposis-colorectal-cancer
IMS: Imaging mass spectrometry
IP: Immunopositivity
MALDI: Matrix-assisted laser desorption/ionization
MMR: DNA mismatch repair system
MSI: Microsatellite instability
nLC: nano liquid chromatography
ROC: Receiver operating characteristic
SPE: Solid phase extraction
TIC: Total ion count
TMA: Tissue microarray
Wnt: Wingless
