# Supplementary material for: Spatial UBE2N protein expression indicates genomic instability in colorectal cancers
Source: BMC Cancer. 2019 Jul 18;19:710. doi: 10.1186/s12885-019-5856-1 (PMC6639966; doi:10.1186/s12885-019-5856-1)
Supplement: Supplementary file 1 — Figure S1. MALDI imaging intensity box plots of identified peptides for UBE2N (m/z 1043.635 & m/z 1203.593; top) and SPTBN1 (m/z 959.513 & m/z 1203.593 bottom). Intensity plots of the two identified peptides UBE2N and SPTBN1 detected differentially regulated by MALDI imaging. Figure S2. Tissue-microarray-based immunohistochemical evaluation of UBE2N by means of image scope comparing normal mucosa vs. CRC and euploid vs. aneuploid CRCs after combining data of the training and validation TMA set. Bar plots and ROC curve of immunhistochemical data of UBE2N after combining data of the training and validation TMA set. Figure S3. Exemplary immunohistochemical UBE2N stainings of the TMA validation set. Images are presented as an overview (A) as well as an image section (B). Overview of exemplary immuohistochemical images of UBE2N. The images present results from the TMA validation set. Figure S4. Tissue-microarray-based immunohistochemical evaluation of SPTBN1 by means of image scope comparing normal mucosa vs. CRC and euploid vs. aneuploid CRCs in the training and validation set. Bar plots of immunhistochemical data of SPTBN1. The images present results from the TMA validation set. (DOCX 21654 kb) [file 12885_2019_5856_MOESM1_ESM.docx]

**Supplemental Data**

**Figure S1**: MALDI imaging intensity box plots of identified peptides for UBE2N (m/z 1043.635 & m/z 1203.593; top) and SPTBN1 (m/z 959.513 & m/z 1203.593 bottom)


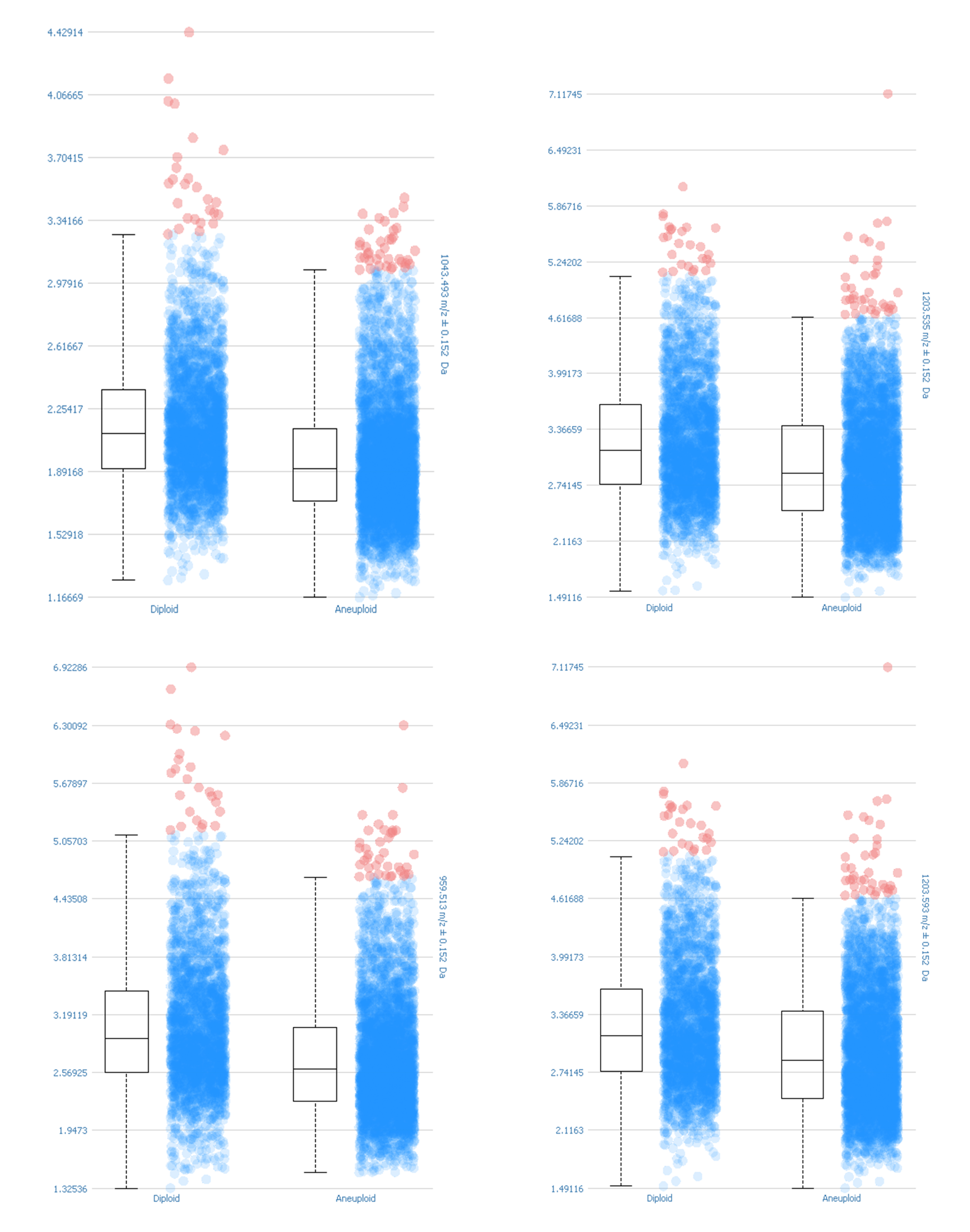


**Figure S2**: Tissue-microarray-based immunohistochemical evaluation of UBE2N by means of image scope comparing normal mucosa vs. CRC and euploid vs. aneuploid CRCs after combining data of the training and validation TMA set. ROC curve (B) represents good discriminative power to distinguish between euploid and aneuploid CRC (**0.001 < P < 0.01, *0.01 < P < 0.05). NM, normal mucosa; CRC, colorectal cancer; IP, immunopositivity; ROC AUC, Receiver operating characteristic area under curve

**
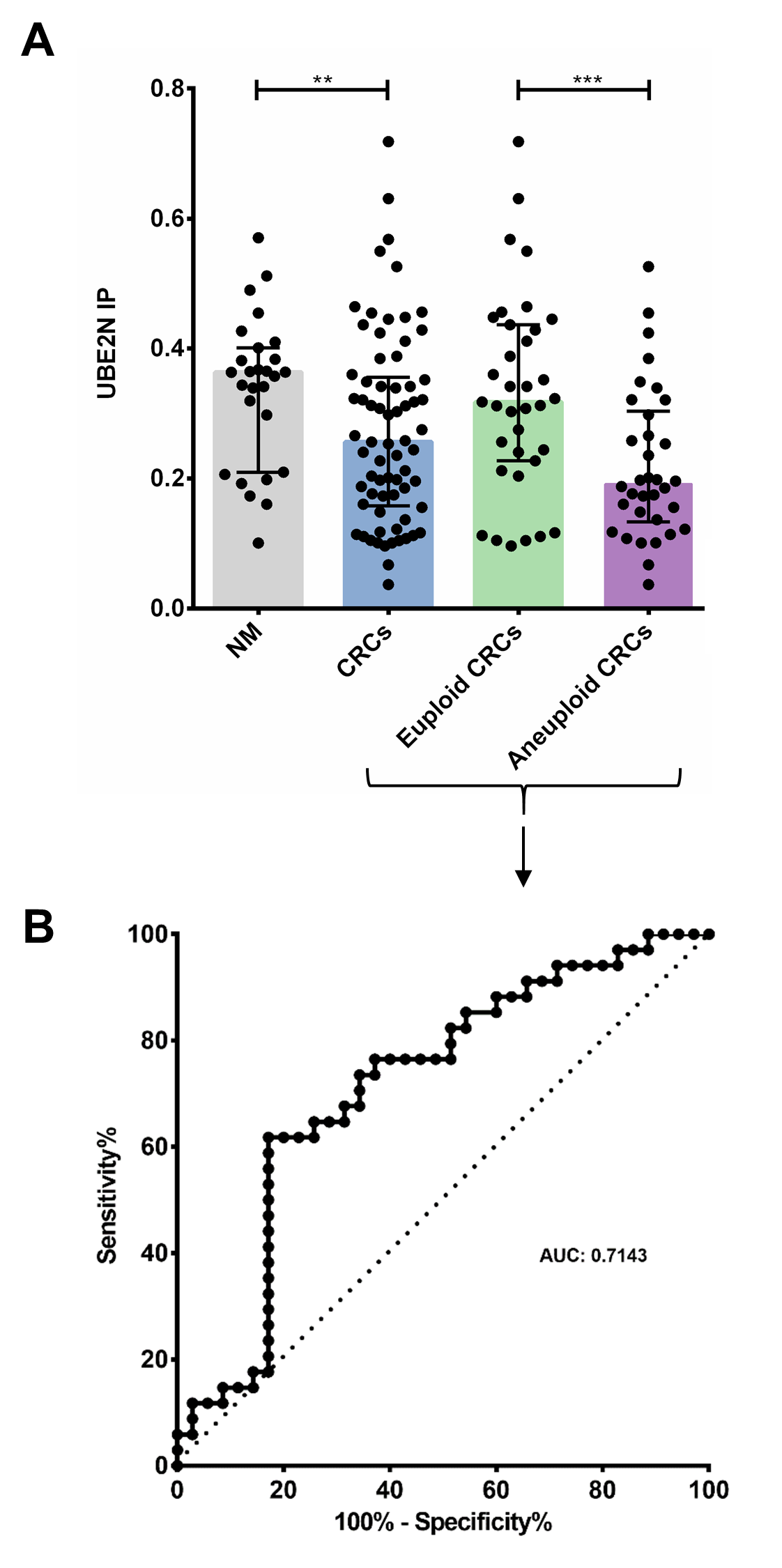
**

**Figure S3**: Exemplary immunohistochemical UBE2N stainings of the TMA validation set. Images are presented as an overview (A) as well as an image section (B). * exemplary samples for the weak and strong images were selected based on low and high immunopositivity values in the individual groups; IP, immunopositivity

**
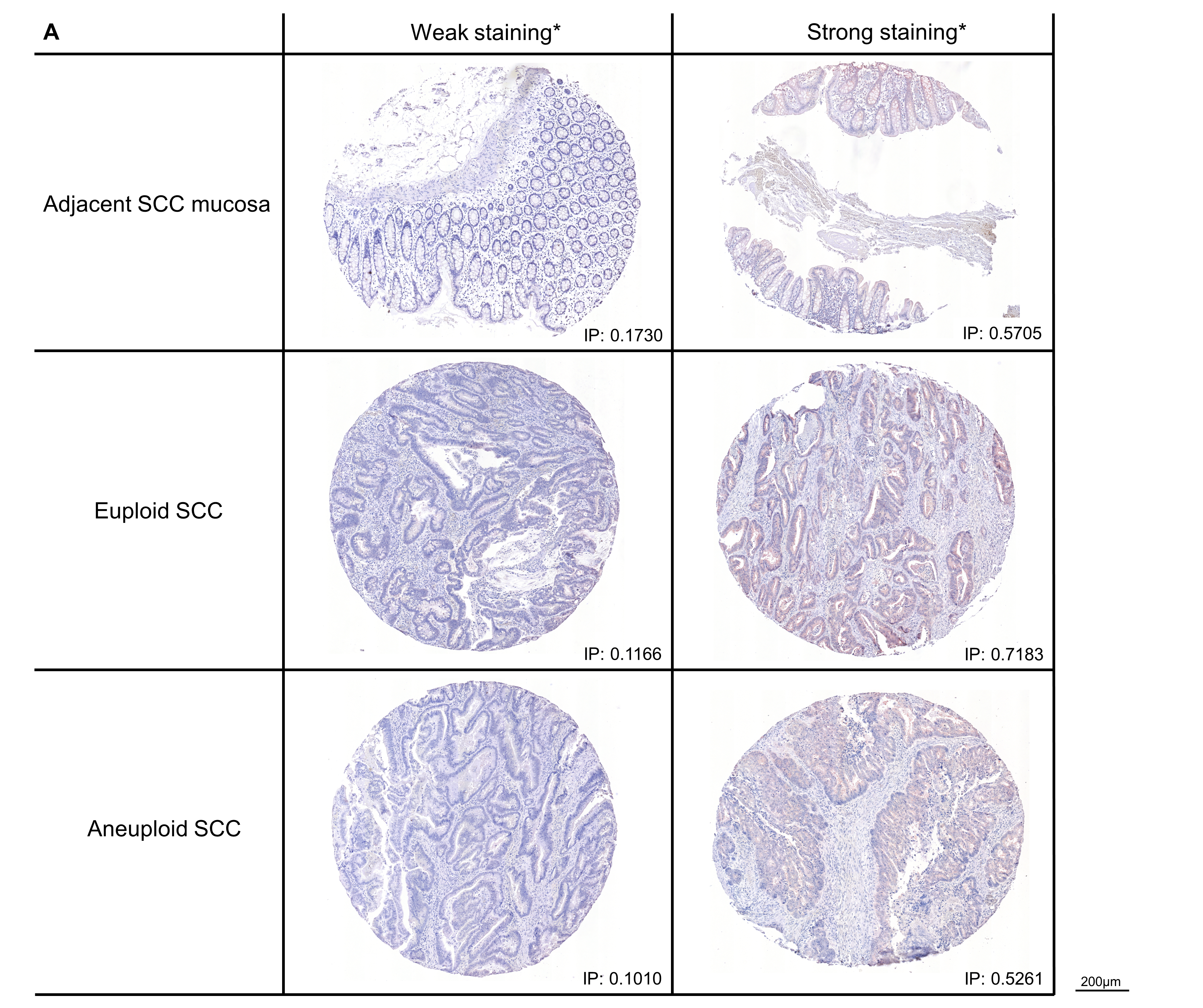
**

**
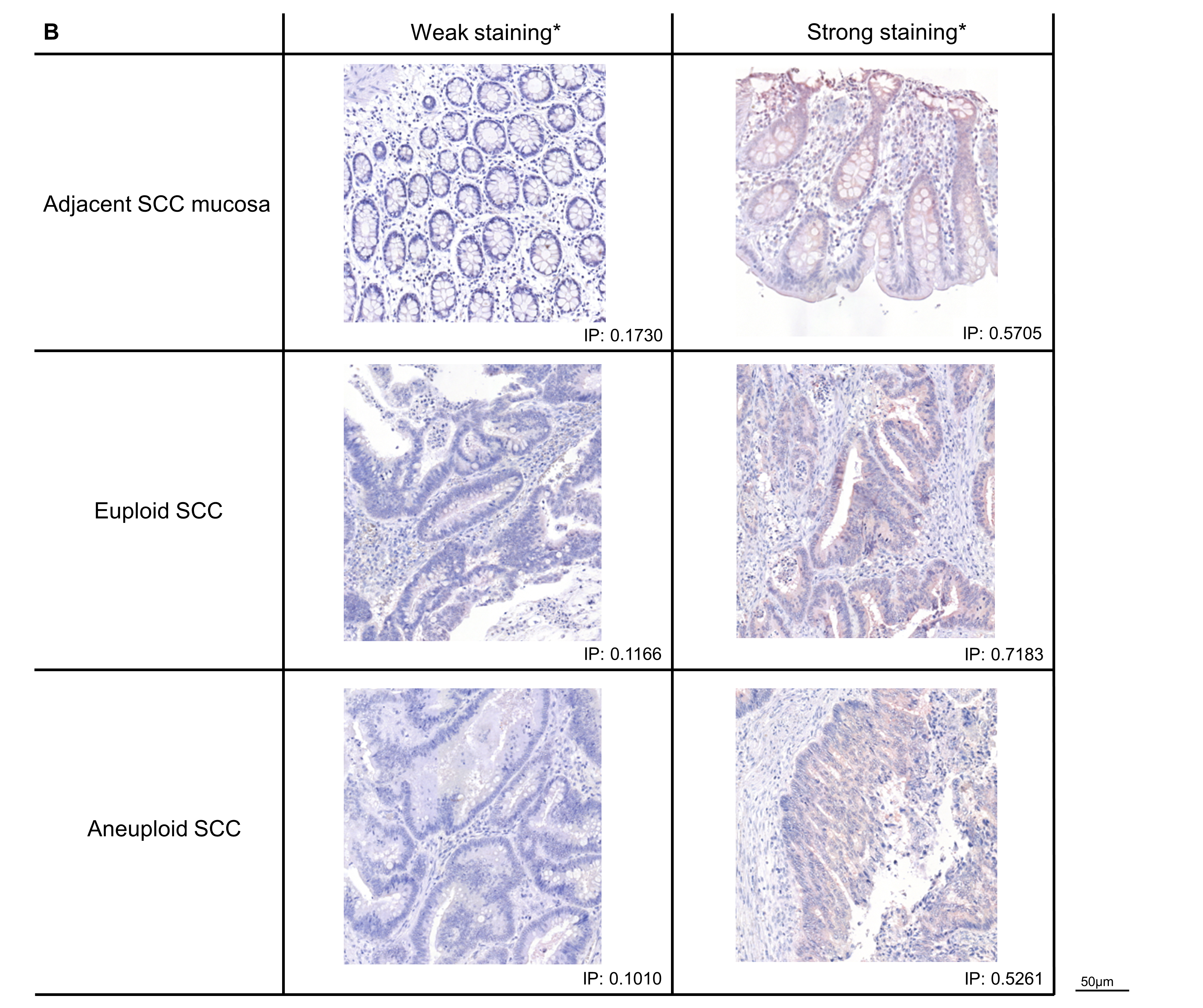
**

**Figure S4**: Tissue-microarray-based immunohistochemical evaluation of SPTBN1 by means of image scope comparing normal mucosa vs. CRC and euploid vs. aneuploid CRCs in the training validation set. NM, normal mucosa; CRC, colorectal cancer; IP, immunopositivity

**

**
